# Eosinophilic granulomatosis with polyangiitis (churg-strauss syndrome) without respiratory symptoms in a boy

**DOI:** 10.1186/1546-0096-12-S1-P366

**Published:** 2014-09-17

**Authors:** Selçuk Yüksel, Havva Evrengül, Tülay Becerir, Ali Koçyiğit, Mine Cinbiş, Neşe Çallı Demirkan

**Affiliations:** 1 Pediatric Rheumatology, Pamukkale University School of Medicine, Denizli, Turkey; 2 Radiodiagnostic, Pamukkale University School of Medicine, Denizli, Turkey; 3 Pediatrics, Pamukkale University School of Medicine, Denizli, Turkey; 4 Pathology, Pamukkale University School of Medicine, Denizli, Turkey

## Introduction

Eosinophilic granulomatosis with polyangiitis or Churg-Strauss Syndrome (CSS) is rare in children. It is characterized by eosinophilia, extravascular necrotizing granuloma, and eosinophilic infiltration of multiple organs particularly lungs, but may also involve the gastrointestinal tract, heart, and the kidneys.

## Objectives

The condition is usually associated with a preceding history of asthma or allergic sinusitis. It has rarely been reported in children, where most of the cases had pre-existing asthma, allergic rhinitis, or atopic disease. We present a boy with CSS, without history of asthma or allergic rhinitis and respiratory symptoms or signs.

## Methods

A fourteen-year old male patient was admitted to our pediatric rheumatology department with a 7 days history of severe extremity pains and generalized body rash. Past medical history revealed that he had no asthma and other allergic diseases, recurring sinusitis, familial Mediterranean fever or vasculitis.

## Results

During physical examination, he was very irritable and agitated due to severe leg pain. Blood pressure, weight, height, respiratory rate and body temperature were normal. He suffered from severe pain in all extremities. Neurological examination revealed 1–2/5 strength in all muscle groups of his lower and upper extremities. He had sensation loss similar to gloves or socks type, and macular rash on the back of hands and soles of feet. Respiratory system and other systems were normal. Laboratory investigations revealed peripheral blood eosinophilia (30%), slight thrombocytopenia (112,000/mm3), high muscle enzymes (CK: 2700U/L, AST: 47U/L, LDH: 249mg/dL), slightly high CRP (1.1mg/dL), IgE (103mg/dL) and ASO (266 IU/mL) levels. All viral, bacterial and parasitological investigations, full urine exam, other biochemical parameters, rheumatologic serological investigations, heavy metal levels (lead and mercury), eye exam, chest X-ray, echocardiography and bone marrow evaluation, abdominal MR and selective renal angiography were normal. Electroneuromyography (ENMG) showed sensorimotor polyneuropathy in all extremities especially in the upper. Skin biopsy revealed necrotizing eosinophilic vasculitis. Thorax MRI showed peripheral infiltration of the anterior segment of the left upper lobe and the patient was diagnosed as CCS. After three days of pulse methylprednisolone, 60 mg/day oral steroid therapy (1 month) was started, then while monthly reduction of steroid continued, IV pulse cyclophosphamide (once a month for 6 months, twice every 3 months) and then azathioprine (2 mg/kg/day), gabapentin, oral vitamin D and calcium treatment were given. Meanwhile physical therapy was commenced. All clinical, laboratory and MRI findings were resolved in the first 2 months of treatment. Polyneuropathy was checked by ENMG every 6 months and noted clear improvement in all extremities. On the last visit of the patient in the 20th month, he noted minimal weakness of left hand muscle associated with minimal hypoesthesia.

## Conclusion

In cases suspected of CSS, even without respiratory symptoms, detailed imaging and pathology will establish the exact diagnosis. As in our case, though all findings regress with intense immunosuppressive treatment in the early period, it takes a long time for neuropathy to resolve completely.

## Disclosure of interest

None declared

